# Chronic hypoxia modulates tumour cell radioresponse through cytokine-inducible nitric oxide synthase

**DOI:** 10.1054/bjoc.2000.1719

**Published:** 2001-04

**Authors:** D L Van den Berge, M De Ridder, V N Verovski, M Y Janssens, C Monsaert, G A Storme

**Affiliations:** Cancer Research Unit, Academic Hospital Free University Brussels, Oncology Center, Laarbeeklaan 101, Brussels, 1090, Belgium

**Keywords:** chronic hypoxia, radiosensitization, nitric oxide synthase, nitric oxide, cytokines

## Abstract

Chronic hypoxia up-regulated the mRNA and protein expression of inducible nitric oxide synthase (iNOS) in EMT-6 tumour cells exposed to interferon (IFN)-gamma and interleukin (IL)-I beta. Low concentrations of cytokines (1 unit ml^−1^) in 1% but not in 21% oxygen induced a remarkable increase in NO production and a 1.8-fold hypoxic cell radiosensitization. Therefore, chronic hypoxia may potentially be exploited to increase tumour cell radioresponse through the cytokine-inducible iNOS pathway. © 2001 Cancer Research Campaign http://www.bjcancer.com


*British Journal of Cancer* (2001) **84**(8), 1122–1125
© 2001 Cancer Research Campaign
doi: 10.1054/ bjoc.2001.1719, available online at http://www.idealibrary.com on IDEAL®

### Chronic hypoxia modulates tumour cell radioresponse through cytokine-inducible nitric oxide synthase

**DL Van den Berge, M De Ridder, VN Verovski, MY Janssens, C Monsaert and GA Storme**

Academic Hospital Free University Brussels, Oncology Center, Cancer Research Unit, Laarbeeklaan 101, 1090 Brussels, Belgium

**Summary** Chronic hypoxia up-regulated the mRNA and protein expression of inducible nitric oxide synthase (iNOS) in EMT-6 tumour cells exposed to interferon (IFN)-gamma and interleukin (IL)-I beta. Low concentrations of cytokines (1 unit $ml^{-1}$) in 1% but not in 21% oxygen induced a remarkable increase in NO production and a 1.8-fold hypoxic cell radiosensitization. Therefore, chronic hypoxia may potentially be exploited to increase tumour cell radioresponse through the cytokine-inducible iNOS pathway. © 2001 Cancer Research Campaign http://www.bjcancer.com

**Keywords**: chronic hypoxia; radiosensitization; nitric oxide synthase; nitric oxide; cytokines

Solid tumours are supplied with lower oxygen levels than normal tissues because of poorly developed vasculature and sporadic occlusions of blood vessels. Severe hypoxia below 0.3% oxygen is frequently registered in melanomas, breast, cervical, head and neck cancers and is thought be the single most important cause of clinical radioresistance (Dachs and Stratford, 1996; Dachs and Tozer, 2000). The hypoxic tumour microenvironment is also responsible for the selection of malignant clones that overexpress hypoxia-inducible genes responsible for tumour angiogenesis. Besides vascular endothelial growth factor, a direct mediator of vascularization, the cytokine-inducible form of nitric oxide synthase (iNOS) appears to respond to a hypoxia stress since its activation is detected in many solid tumours (Thomsen et al, 1994, 1995; Ambs et al, 1998; Thomsen and Miles, 1998). This enzyme utilizes L-arginine to produce the nitric oxide radical (NO) that promotes tumour growth at low concentrations (Wink et al, 1998; Dachs and Tozer, 2000). However, as we have recently demonstrated in EMT-6 tumour cells, the induction of iNOS by cytokines in aerobic conditions can trigger an NO production high enough to reverse hypoxia-induced radioresistance (Janssens et al, 1998). The question of how this mechanism is influenced by chronic hypoxia is essential and may determine whether it can be exploited as a novel hypoxia-selective target.

The present study is a first step in this direction and explores the possibility that chronic hypoxia may modulate hypoxic cell radioresponse through up-regulation of iNOS. We asked whether hypoxia alone may cause iNOS induction or whether cooperation with cytokines is required to achieve full iNOS expression. Secondly, we examined the activity of iNOS after chronic hypoxia and evaluated NO-mediated hypoxic cell radiosensitization.

*Received 24 October 2000*
*Revised 19 January 2001*
*Accepted 23 January 2001*

*Correspondence to:* DL Van den Berge

### MATERIALS AND METHODS

### Chemicals

All reagents were obtained from Sigma Chemical Co (St. Louis, MO, USA) unless otherwise stated.

### Cell culture

Murine mammary adenocarcinoma EMT-6 cells were kindly provided by Dr Edith Lord (University of Rochester, Cancer Center, New York, USA). The cells (passages 15–40) were propagated in RPMI 1640 medium (Gibco, Paisley, United Kingdom) supplemented with 10% bovine calf serum (HyClone Laboratories Inc Logan, Utah, USA) in plastic flasks (Greiner, Frickenhausen, Germany).

### iNOS induction by cytokines

EMT-6 monolayer cultures grown to early confluence were exposed to IL-1β plus IFN-γ for 16 h in normoxia or hypoxia. To obtain chronic hypoxia, culture flasks were placed in sealed chambers and subjected to repeated vacuum evacuation/injection of nitrogen/$CO_2$-balanced gas containing 0.03 or 1% oxygen. Further processing of cells was done as described below.

### Western and Northern blot analysis

After iNOS induction, the protein and mRNA levels of iNOS were estimated by Western and Northern blotting as described previously (Janssens et al, 1998). Briefly, lysates from $1 \times 10^5$ cells were resolved in a 7.5% polyacrylamide-SDS gel, blots were stained with the monoclonal antibody to iNOS (Affiniti Research Products, Exeter, UK) and analysed by an immunoperoxidase-based ECL technique (Amersham). RNA samples (10 μg) were electrophoresced on formaldehyde agarose gels, transferred to HyBond-N membranes (Amersham) and probed with $^{32}P$-labelled 1.8-kb mouse iNOS cDNA fragment (Alexis Corporation, Laufelfingen, Switzerland).

### Amperometric measurement of nitric oxide

After iNOS induction, amperometric measurements of NO were performed in cell suspensions ($30 \times 10^6$ ml$^{-1}$, 37˚C) in ambient atmosphere. The NO signal was registered by an Iso-NOP200 microsensor (World Precision Instruments, Hertfordshire, UK) and data were expressed in nA (Janssens et al, 1998).

### Determination of nitrite

After iNOS induction, cultures were washed out from cytokines and re-incubated during 24 h in normoxia to accumulate nitrite, an oxidative product of NO. The nitrite level in the medium was determined using the Griess reaction as described elsewhere (Titheradge, 1998).

### Radiosensitivity

After iNOS induction, the cells were harvested from cultures by trypsinization and micropellets ($0.5 \times 10^6$ cells) were produced in conical tubes by centrifugation at 300 ***g*** for 5 min. Metabolic oxygen depletion in micropellets was induced by incubation at 37˚C for 3 min prior to radiation. Using this procedure for various mouse and human tumour cells, we have previously found oxygen enhancement ratios in the range of 2.5–3.0 indicating radiobiologically relevant hypoxia (Verovski et al, 1996; Janssens et al, 1998, 1999). Micropellets were irradiated with a linear accelerator at a rate of 2 Gy per min and survival after 4, 8, 12 and 16 Gy was measured by a 8-day colony formation assay. Radiosensitization was evaluated at the level of 0.1 surviving fraction.

### Statistics

All assays were repeated at least 3 times. Data are expressed as means (symbols, columns) with corresponding standard deviations (SD, bars).

### RESULTS

EMT-6 cultures were exposed to IFN-γ plus IL-1β for 16 h in varying oxygen concentrations and afterwards cells were analysed for iNOS expression and production of nitrite, an oxidized metabolite of NO. Normoxia (21% oxygen) was compared to 1% and 0.03% oxygen, respectively modeling reduced oxygenation in tumour tissue and radiobiologically relevant hypoxia.

In aerobic cells, Western blots demonstrated activation of iNOS expression at 3–10 units ml$^{-1}$ cytokines while in the absence of cytokines no iNOS protein was found (Figure 1). In 0.03 and 1% oxygen, cytokines substantialy up-regulated iNOS expression that became evident at 0.3–1 units ml$^{-1}$. The hypoxic up-regulation of iNOS by cytokines resulted in appearance of active enzyme since the accumulation of nitrite in the medium at 0.3–1 units ml$^{-1}$ was increased over the next 24 h (Figure 2). A low but detectable level of iNOS protein and activity was found in the samples exposed to hypoxia without cytokines. Northern blot data were in agreement with the protein expression and suggested a transcriptional up-regulation of iNOS induction in hypoxia (Figure 1). There was some increase in iNOS up-regulation in 0.03% compared with 1% oxygen but deep hypoxia during 16 h caused cytotoxicity. Therefore, further experiments were conducted in 1% oxygen.

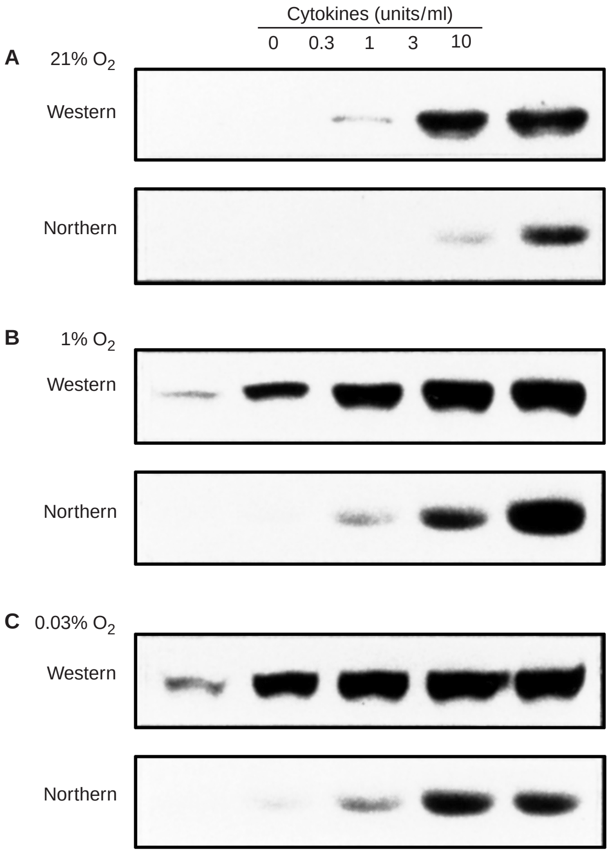

**Figure 1** Western and Northern blot analysis of iNOS protein and mRNA expression in EMT-6 cells after a 16 h exposure to IFN-γ and IL-1β in 21% (**A**), 1% (**B**) or 0.03% (**C**) oxygen

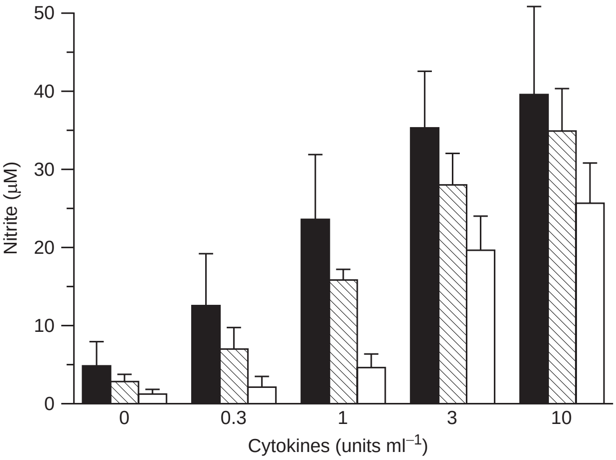

**Figure 2** Nitrite production in EMT-6 cells after a 16 h exposure to IFN-γ and IL-1β in 21% (□) 1% (▧) or 0.03% (■) oxygen. The nitrite was accumulated in the medium during a 24 h re-incubation period and determined by the Griess reaction

To measure NO output, concentrated cell suspensions were prepared from EMT-6 cultures treated or not with cytokines, and the NO signal was registered at 37˚C using a NO-specific microsensor (Figure 3). At 1 unit ml$^{-1}$, hypoxic but not aerobic treatment resulted in a substantial NO signal in line with the blot

© 2001 Cancer Research Campaign

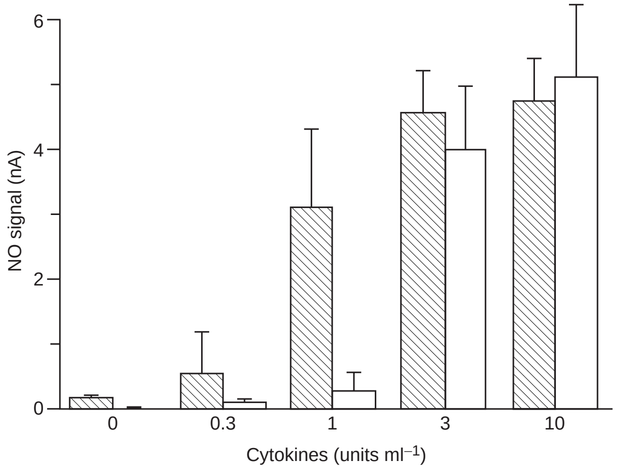

**Figure 3** NO production in EMT-6 cells after a 16 h exposure to IFN-γ and IL-1β in 21% (□) or 1% (▨) oxygen. The NO signal was registered by a NO-specific microsensor in concentrated cell suspensions ($30 \times 10^6$ ml$^{-1}$)

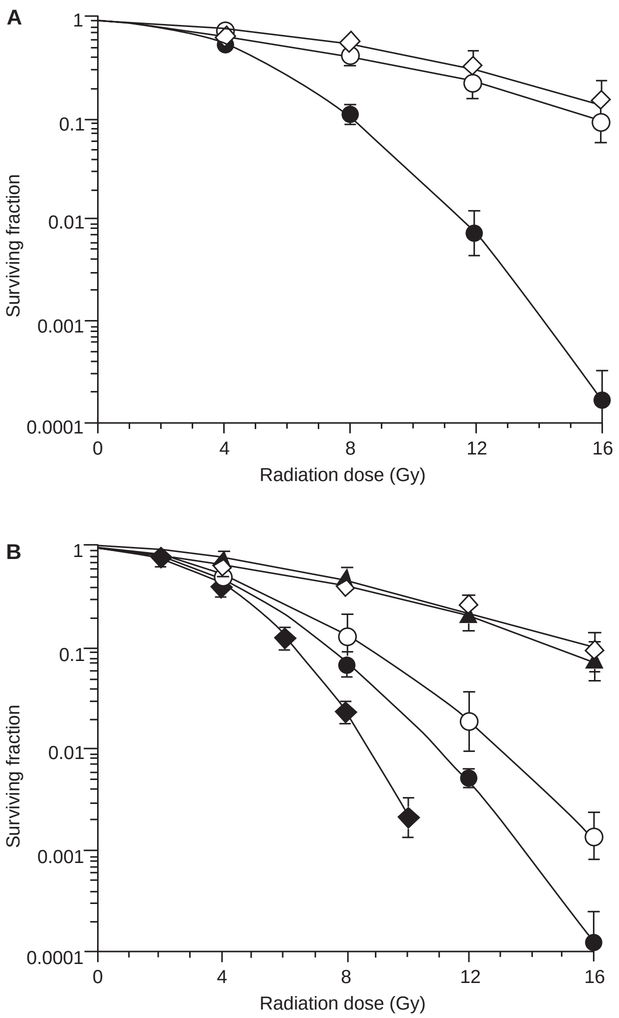

**Figure 4** Radiosensitivity of EMT-6 cells after a 16 h exposure to IFN-γ and IL-1β in 21% (**A**) or 1% (**B**) oxygen. EMT-6 cells, not treated (◇) or treated with cytokines at 1 (○) or 3 (●) units ml$^{-1}$, were isolated and analysed for hypoxic cell radiosensitivity in micropellets. The iNOS inhibitor aminoguanidine (1 mM) was added 1 h prior to cell isolation (▲). The survival curve for aerobic cells (◆) is plotted for reference and Griess data (Figures 1 and 2). In absence of cytokines, only a background level of NO production was observed for both treatments. At cytokine concentrations higher than 1 units ml$^{-1}$, comparable iNOS activity was found in hypoxia and normoxia.

We have already demonstrated iNOS-induced radiosensitization in cell micropellets subjected to metabolic oxygen depletion, and identified L-arginine-derived NO as a radiosensitizer (Janssens et al, 1998). The same model was applied to verify the increased radiosensitizing potency of cytokines under hypoxic versus aerobic conditions. As shown in Figure 4A, the radiosensitivity of EMT-6 cells exposed to 1 unit ml$^{-1}$ cytokines in 21% oxygen was unchanged. Strikingly, the same concentration of cytokines in 1% oxygen induced a 1.8-fold radiosensitization (Figure 4B). A raised concentration of cytokines (3 units ml$^{-1}$) was active under both hypoxic and aerobic conditions and caused comparable radiosensitization (2.1 to 2.3-fold) approaching the radiosensitizing effect of oxygen (2.5-fold). In the absence of cytokines, chronic hypoxia only marginally altered hypoxic cell radioresponse in micropellets. The specificity of iNOS induction in chronic hypoxia was confirmed with the iNOS inhibitor aminoguanidine that abrogated the radiosensitizing effect of cytokines (Figure 4B).

### DISCUSSION

This study shows that chronic hypoxia in the range of 0.03–1% oxygen increases the potency of cytokines IL-1β and IFN-γ to activate iNOS in EMT-6 tumour cells. In comparison to aerobic cells, hypoxic cells revealed substantially up-regulated levels of iNOS protein when exposed to low concentration of cytokines (0.3–1 units ml$^{-1}$). In the absence of cytokines, no iNOS was found in aerobic cells while hypoxic cells expressed iNOS at a low but detectable level. Compared to 1% oxygen, the radiobiologically relevant hypoxia level of 0.03% oxygen further potentiated cytokine effects as suggested by nitrite accumulation and protein expression. Activation of iNOS in hypoxic EMT-6 cells was controlled at the transcriptional level since protein expression reflected mRNA levels. Transcriptional up-regulation of iNOS in moderate hypoxia (1% oxygen) was previously shown in human hepatocellular carcinoma Hep3B cells (Yoshioka et al, 1997) but was absent in human intestinal adenocarcinoma DLD-1 cells (Salzman et al, 1996). While synergism of cytokines and hypoxia in iNOS induction is well established for macrophages (Melillo et al, 1995) such an interaction may not always be present in tumour cells that display different profiles of cytokine receptors and hypoxia-inducible transcription factors.

Although activation of iNOS in both stromal and tumour cells is described for many malignancies (Thomsen et al, 1994, 1995; Ambs et al, 1998; Thomsen and Miles, 1998), the level and role of NO in tumour physiology are not clear. A growing body of evidence suggests that NO production in tumour occurs at a low rate and provides primarily a positive growth signal (Thomsen and Miles, 1998; Wink et al, 1998; Dachs and Tozer, 2000). To achieve radiosensitization, much higher NO concentrations may be required as show our studies with NO donors (Verovski et al, 1996; Janssens et al, 1999) and other reports (Griffin et al, 1996; Mitchell et al, 1996). However no obvious correlation between

 *© 2001 Cancer Research Campaign*

NO levels and radiosensitization was found and NO produced intracellularly in the proximity of radiation targets is likely to be more effective than chemical NO releasers. Indeed, we demonstrated that iNOS generates a 10-times lower NO output compared to that of the NO donor PAPA/NO yet resulting in comparable radiosensitization (Janssens et al, 1998).

Therefore, we raised the question whether cytokine-induced iNOS activation in chronic hypoxia results in sufficient NO production to achieve radiosensitization. Secondly, we asked whether chronic hypoxia may increase the radiosensitizing potency of cytokines that would be consistent with up-regulated profiles of iNOS expression in hypoxia. We have measured NO output in EMT-6 cells using a NO-specific sensor and found a remarkable increase in NO signal induced by 1 unit $ml^{-1}$ cytokines in hypoxia but not in normoxia. Likewise, selective induction of iNOS in hypoxia resulted in significant radiosensitization (1.8-fold) that could be abrogated by the iNOS inhibitor aminoguanidine. In contrast, aerobic cytokine treatment was not effective at 1 unit $ml^{-1}$, and chronic hypoxia alone had little effect on radiosensitivity. Taken together, these data suggest that chronic hypoxia does up-regulate iNOS expression in tumour cells but that it sustains rather than triggers iNOS activation. Hence, cytokines continue to play a crucial role in iNOS activation and iNOS-mediated radiosensitization. Whether this mechanism is operational in human tumour cells and which tumours overexpress iNOS in hypoxia remains to be explored.

In conclusion, chronic hypoxia up-regulates cytokine-induced expression of iNOS in EMT-6 tumour cells resulting in appearance of functionally active enzyme. Therefore hypoxia may potentially be exploited to increase the radiosensitizing activity of cytokines through the iNOS pathway.

### ACKNOWLEDGEMENTS

The authors are grateful to Prof Dr M Mareel and Prof Dr K Thielemans for expert assistance.

This research was funded by grants nrs. G.0055.96 and G.0195.98 from the Fonds voor Wetenschappelijk Onderzoek-Vlaanderen and Sportvereniging tegen Kanker and Vlaamse Kankerliga.

### REFERENCES

Ambs S, Merriam WG, Bennett WP, Felley-Bosco E, Ogunfusika MO, Oser SM, Klein S, Shields PG, Billiar TR and Harris CC (1998) Frequent nitric oxide synthase-2 expression in human colon adenomas: implication for tumor angiogenesis and colon cancer progression. *Cancer Res* **58**: 334–341

Dachs GU and Stratford IJ (1996) The molecular response of mammalian cells to hypoxia and the potential for exploitation in cancer therapy. *Br J Cancer* **74**: (Suppl XXVII) S126–S132

Dachs GU and Tozer GM (2000) Hypoxia modulated gene expression: angiogenesis, metastasis and therapeutic exploitation. *Eur J Cancer* **36**: 1649–1660

Griffin RJ, Makepeace CM, Hur WJ and Song CW (1996) Radiosensitization of hypoxic tumor cells in vitro by nitric oxide. *Int J Rad Oncol Biol Phys* **36**: 377–383

Janssens MY, Van den Berge DL, Verovski VN, Monsaert C and Storme GA (1998) Activation of inducible nitric oxide synthase results in nitric oxide-mediated radiosensitization of hypoxic EMT-6 tumor cells. *Cancer Res* **58**: 5646–5648

Janssens MY, Verovski VN, Van den Berge DL, Monsaert C and Storme GA (1999) Radiosensitization of hypoxic tumour cells by S-nitroso-N-acetylpenicillamine implicates a bioreductive mechanism of nitric oxide generation. *Br J Cancer* **79**: 1085–1089

Melillo G, Musso T, Sica A, Taylor LS, Cox GW and Varesio L (1995) A hypoxia-responsive element mediates a novel pathway of activation of the inducible nitric oxide synthase promoter. *J Exp Med* **182**: 1683–1693

Mitchell JB, Cook JA, Krishna MC, DeGraff W, Gamson J, Fisher J, Christodoulou D and Wink DA (1996) Radiation sensitisation by nitric oxide releasing agents. *Br J Cancer* **74**: (Suppl XXVII) S181–S184

Salzman A, Denenberg AG, Ueta I, O'Connor M, Linn SC and Szabom C (1996) Induction and activity of nitric oxide synthase in cultured human intestinal epithelial monolayers. *Am J Physiol* **270**: G565–G573

Thomsen LL and Miles DW (1998) Role of nitric oxide in tumour progression: lessons from human tumours. *Cancer Metastasis Rev* **17**: 107–118

Thomsen LL, Lawton FG, Knowles RG, Beesley JE, Riveros-Moreno V and Moncada S (1994) Nitric oxide synthase activity in human gynecological cancer. *Cancer Res* **54**: 1352–1354

Thomsen LL, Miles DW, Happerfield L, Bobrow LG, Knowles RG and Moncada S (1995) Nitric oxide synthase activity in human breast cancer. *Br J Cancer* **72**: 41–44

Titheradge MA (1998) Enzymatic measurement of nitrate and nitrite. In: Titheradge MA (ed) *Nitric oxide protocols* pp 83–92. Humana P: New Jersey

Verovski VN, Van den Berge DL, Soete GA, Bols BL and Storme GA (1996) Intrinsic radiosensitivity of human pancreatic tumour cells and the radiosensitising potency of the nitric oxide donor sodium nitroprusside. *Br J Cancer* **74**: 1734–1742

Wink DA, Vodovotz Y, Laval J, Laval F, Dewhirst MW and Mitchell JB (1998) The multifaceted roles of nitric oxide in cancer. *Carcinogenesis* **19**: 711–721

Yoshioka K, Thompson J, Miller MJ and Fisher JW (1997) Inducible nitric oxide synthase expression and erythropoietin production in human hepatocellular carcinoma cells. *Biochem Biophys Res Commun* **232**: 702–706

© 2001 Cancer Research Campaign 
